# Measures to Prevent Infection in Cardiac Implantable Electronic Device Replacements or Upgrades

**DOI:** 10.31083/j.rcm2501019

**Published:** 2024-01-10

**Authors:** Carolina Hoyos, Xiaoxiao Qian, Carlos D. Matos, Mohamed Gabr, Daniela Hincapie, John B. Cadigan, Nathaniel Steiger, Juan C. Diaz, William Sauer, Jorge E. Romero

**Affiliations:** ^1^Cardiac Arrhythmia Service, Division of Cardiovascular Medicine, Brigham and Women’s Hospital, Harvard Medical School, Boston, MA 02115, USA; ^2^Cardiac Arrhythmia Center, Montefiore-Einstein Center for Heart and Vascular Care, Division of Cardiology, Department of Medicine, Albert Einstein College of Medicine, Bronx, NY 10467, USA; ^3^Department of Medicine, Tulane University School of Medicine, New Orleans, LA 70112, USA; ^4^Electrophysiology and Cardiac Arrhythmia Service, Clinica Las Vegas, Universidad CES School of Medicine, 050022 Medellin, Colombia

**Keywords:** CIED infection, endocarditis, preventive measures for CIED infection, device upgrades, device replacements

## Abstract

Cardiac implantable electronic device (CIED) infections represent one of the most threatening complications associated with device implantation, due to an increase in morbidity and mortality rates, as well as healthcare costs. Besides, it is important to highlight that when compared to the initial implantation of a device, the risks associated with procedures like generator changes, lead and pocket revisions, or device upgrades double. Consequently, to address this issue, various scoring systems, like the PADIT (Prior Procedures, Age, Depressed Renal Function, Immunocompromised Status, Type of Procedure), the RI-AIAC (Ricerca Sulle Infezioni Associate a ImpiAnto o Sostituzione di CIED), and the Shariff score, along with predictive models, have been developed to identify patients at a greater risk of infection. Moreover, several interventions have been assessed to evaluate their role in infection prevention ranging from improving skin preparation and surgical techniques to considering alternative strategies such as the subcutaneous Implantable Cardioverter-Defibrillator (ICD). Methods like antimicrobial prophylaxis, pocket irrigation, chlorhexidine gluconate pocket lavage, capsulectomy, and the use of antibacterial envelopes have been also explored as preventive measures. In this review, we provide a comprehensive assessment of CIED infections in patients undergoing repeat procedures and the strategies designed to reduce the risk of these infections.

## 1. Introduction

**Fig. 1.**
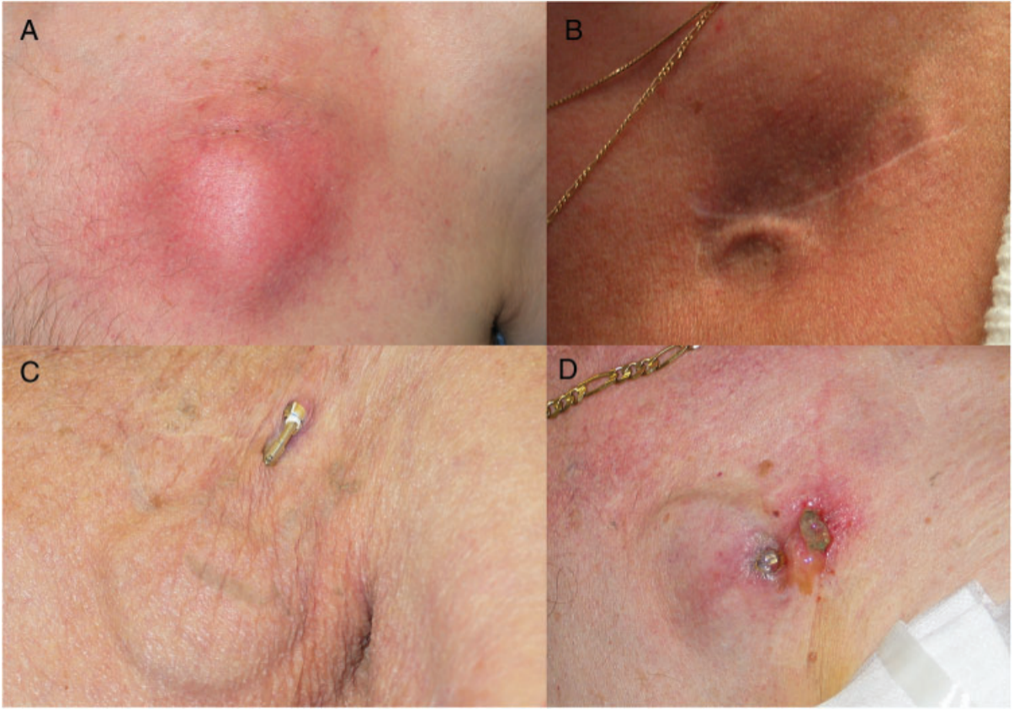
**Examples of CIED infections**. (A) Localized pocket infection. (B) Device tethering consistent with pre-erosion. (C) Device erosion without site inflammation. (D) Localized inflammation and erosion. Reprinted from the Europace article, Han *et al*. [1]. Epidemiology of cardiac implantable electronic device infections: incidence and risk factors, with permission from the Europace journal. Abbreviations: CIED, cardiac implantable electronic device.

Cardiac implantable electronic device (CIED) infection remains one of the most dreaded complications associated with device implantation, leading to increased morbidity, mortality, and healthcare costs. Their severity ranges from a localized pocket inflammation to localized infection, skin erosion with risk of sepsis, or systemic and bloodstream infection [1] (Fig. 1, Ref. [1]). Compared to de novo implantation, the risks associated with generator changes, lead or pocket revisions, or upgrades are further increased, with prior studies demonstrating up to a 2.2-fold increased risk of pocket-related complications requiring re-intervention [2]. The REPLACE registry, for instance, previously demonstrated higher rates of complications in patients undergoing generator replacement with additional lead upgrade or replacement compared to those without [3]. Consequently, numerous scores and prediction models have been developed to identify patients at higher risk of infection, and various interventions have been assessed to evaluate their role in infection prevention. In this review, we systematically assess the incidence of CIED infections in patients undergoing repeat procedures, concurrently examining a range of strategies devised to mitigate and manage this associated risk.

## 2. Incidence and Epidemiology

The incidence of CIED infections increased from 1.61% in 1993 to 2.41% in 2008 [4], owing its augmenting rate to the growing implantation of more complex devices in older patients with more comorbidities [5]. The National Inpatient Sample (NIS), a 12-year follow-up study (2000–2012) that included 4,144,683 CIED procedures, showed a CIED infection rate of 2.06% [6]. In the interim, a Danish registry which included 97,750 patients from 1982 to 2018, demonstrated a CIED infection rate of 1.19% for permanent pacemakers (PPM) and 3.35% for Cardiac Resynchronization Therapy - Defibrillators (CRT-D) [5]. Furthermore, a more recent study published in Europace in 2023 by Modi V *et al*. [7], which analyzed 1,604,173 admissions for CIED implantations among the NIS between 2011 and 2018, reported the highest incidence of CIED infections at a rate of 4.4%. This study found no significant variation in the annual admission rates for CIED infections (ranging from 3.96% to 4.59%, *p*-value = 0.98) and identified predisposing factors such as male gender, non-white race, and low-income status. The in-hospital mortality rate among patients admitted with CIED infection was 4.3%, which is comparatively low when compared to previous registries from the Medicare fee-for-service database, where rates ranged from 5% to 8% [7]. It is noteworthy that patients admitted for CIED infection had an increased prevalence of congestive heart failure (CHF), which rose from 50% in 2011 to 64.4% in 2018. The most frequently observed complications were pulmonary embolism (4.1%), deep vein thrombosis (3.6%), and post-procedural hematoma (2.9%) [7]. Moreover, a multivariate analysis revealed that CHF (odds ratio [OR] 1.67; 95% confidence interval [CI] = 1.35–2.07), end-stage renal disease (ESRD) (OR 1.90; 95% CI = 1.46–2.48), coagulopathy (OR 2.94; 95% CI = 2.40–3.61), and malnutrition (OR 2.50; 95% CI = 1.99–3.15) were identified as predictors of in-hospital mortality in patients admitted for CIED infection [7].

## 3. Pathogenesis

CIED and lead-related infections primarily result from the introduction and subsequent colonization of pathogens during the implantation process. These pathogens can colonize the device pocket, travel along the lead’s path via the venous system, and ultimately result in bacteremia and infectious endocarditis [8]. Predominantly, the organisms isolated in CIED infections belong to the *Staphylococcal genus*, with coagulase-negative microbes prevailing. Importantly, methicillin-resistant *Staphylococcus aureus* constitutes a substantial proportion of Staph-related CIED infections [9]. In addition to *Staphylococci*, Gram-negative organisms, anaerobes, fungi, and mycobacteria have also been identified in cases of CIED infection. However, it is noteworthy that in the early stages, endovascular infections are primarily attributed to *Staphylococcus aureus * [9]. An additional source of infection in the intravascular components of CIED may stem from secondary contamination through vascular catheters or from infections originating distally at other sites such as the urinary, hepatobiliary, or respiratory systems, also known as seeding [10].

The pathogenesis of CIED infections hinges on the intricate interactions between the microbes, the host, and the device itself. These interactions exhibit variability across different microbe species. Notably, organisms like *Staphylococcus epidermidis* and *Staphylococcus aureus* are part of the normal human microbiota, which raises concerns about their potential introduction during the implantation process [11]. It is worth highlighting that CIED manipulation before implantation and introduction through the incision site are plausible routes of contamination [11]. *Staphylococcus epidermidis*exhibits a two-step adherence process to devices. This entails an initial hydrophobic, non-specific attachment followed by the accumulation and proliferation of the organism, culminating in the formation of a biofilm [11]. This biofilm, essentially an extracellular polysaccharide matrix, serves as a protective shield for the organism against host defenses and stands as a pivotal virulence factor for these microbes. In contrast, *Staphylococcus aureus* is thought to utilize host tissue ligands to adhere to the CIED and subsequently form biofilms [12].

## 4. Predisposing Factors

### 4.1 Host Factors

Multiple patient-related factors, including ESRD, CHF, diabetes mellitus (DM), chronic obstructive pulmonary disease (COPD), immunosuppression, skin disorders, and malignancy, have been identified to be associated with an increased risk for CIED infection [13]. Notably, ESRD and renal insufficiency consistently emerge as significant major risk factors in several studies [14, 15, 16]. Moreover, uremia has been described as a predictor of CIED infection (12.5% *vs.* 0.2%; *p* < 0.0001) and bleeding (21.9% *vs.* 3.2%; *p* < 0.0001) when compared to patients with normal renal function, which is attributed to its impact on the immune system and platelet physiology [14]. Optimization of patient’s comorbidities may decrease the risk of CIED infections. For instance, the proper control of DM with a blood glucose target level of less than 150 mg/dL was reported to be associated with a decreased risk of surgical site infection [17]. Additionally, although immunocompromised patients are frequently excluded from randomized clinical trials as they represent a highly vulnerable population, a retrospective cohort study conducted at the MD Anderson Cancer Center in Houston, Texas demonstrated the benefit of a “comprehensive prophylactic bundle approach”. This approach encompassed preprocedural intravenous vancomycin and intraoperative surgical pocket irrigation with polymyxin B and bacitracin, followed by implantation of the TYRX™ antimicrobial mesh and postprocedural oral minocycline 100 mg twice daily for 5 days. The study demonstrated a non-increased risk of CIED infections within an oncologic population, including patients with solid and hematologic tumors. As such, it was concluded that there is no compelling justification for withholding the placement of a CIED from oncological patients solely on the grounds of infection risk [18].

### 4.2 Procedural Factors

The greatest predisposing factor for CIED infection is reintervention. As demonstrated in an Implantable Cardioverter-Defibrillator (ICD) registry from 2006 to 2009, device upgrade or generator change compared to initial implant was associated with a significantly higher risk of CIED infection (1.9% *vs*. 1.6%; *p* < 0.0001) [19]. Noticeably, early reintervention (within 30 days) was related to the highest risk of CIED infection [19]. Moreover, the BRUISE-CONTROL randomized controlled trial (RCT) reported that hematoma formation resulted in a 7-fold increased risk of infection within 1-year follow-up [20]. Similarly, an analysis among the patients from the WRAP-IT trial demonstrated a hazard ratio (HR) of infection of 11.3 (95% CI = 5.5–23.2) in patients with hematoma formation [21]. Regarding the type of device, the implantation of ICD or cardiac resynchronization therapy (CRT) is associated with a higher risk of infection compared to PPM, probably related to the complexity of the procedure and the number of leads [5]. Furthermore, the adherence of microorganisms to the device is influenced by the intrinsic properties of the CIED. Irregular hydrophobic surfaces and synthetic sources are more favorable for microorganism adhesion [22]. Likewise, some materials have a greater propensity for bacterial adherence such as stainless steel and polyethylene, when contrasted to titanium and polyurethane, respectively [22].

## 5. Risk Stratification

Numerous studies have proposed risk scores that incorporate patient-related factors, device characteristics, and specific procedure types for risk stratification. While none of these risk scores have been formally integrated into the current guidelines as part of the standard practice, they do serve as valuable tools in clinical practice by identifying patients at high risk and guiding the implementation of additional preventive measures beyond the conventional sterile precautions.

### 5.1 PADIT (Prior Procedures, Age, Depressed Renal Function, Immunocompromised Status, Type of Procedure) Risk Score

**Fig. 2.**
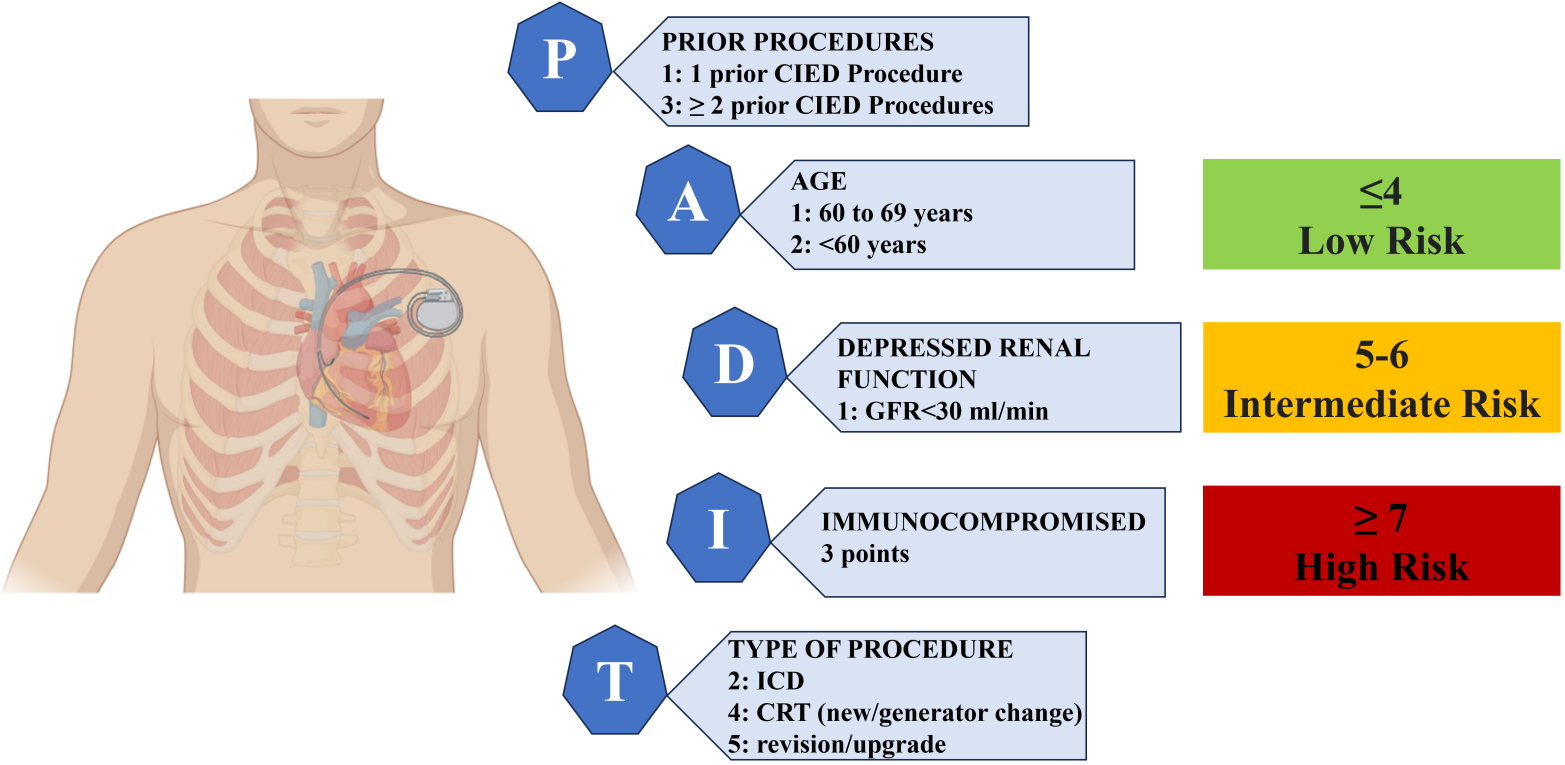
**The PADIT risk score includes five independent predictors of device infection: P: prior procedures, A: age, D: depressed renal function, I: immunocompromised status, and T: procedure type, classifying the patient in low, intermediate, and high risk for CIED infection**. Immunocompromised status was defined as individuals who were either undergoing therapeutic interventions that suppress their innate resistance to infections, such as immunosuppressive treatments, chemotherapy, radiotherapy, and prolonged or recent high-dose steroids; or individuals afflicted by pathologies like leukemia, lymphoma, or HIV infection. Abbreviations: CIED, cardiac implantable electronic devices; GFR, glomerular filtration rate; ICD, implantable cardioverter defibrillator; CRT, cardiac resynchronization therapy; HIV, human immunodeficiency virus.

In 2019, Birnie *et al*. [23] developed the PADIT score based on data from the PADIT trial, a prospective multicenter double-blinded study involving 19,603 patients. The PADIT risk score comprises five independent predictors of device infection: prior procedures [P], age [A], depressed renal function [D], immunocompromised status [I], and procedure type [T]. Each of these risk factors is assigned a specific score, and the cumulative scores indicate the overall risk of infection. Patients were categorized into low-risk (0 to 4 points), intermediate-risk (5 to 6 points), and high-risk (≥7 points) groups, with corresponding rates of hospitalization for infection at 1-year follow-up, being 0.51%, 1.42%, and 3.41%, respectively [23] (Fig. 2). The performance of this score was internally and independently validated using a dataset from U.S. health claims [24]. Notably, among all the risk factors incorporated into the score, undergoing pocket revision and device upgrade posed the highest risk of device-related infection (OR 4.16; 95% CI = 2.74–6.32), and as expected, having a history of more than one previous procedure is also a strong predictor of infection (OR 3.37; 95% CI = 2.11–5.39) [23].

### 5.2 PACE DRAP Score

One limitation of the PADIT prediction model is its lack of consideration of the patient’s bleeding risk, as data on perioperative management of anticoagulation and antiplatelet therapy were not collected in the PADIT trial [23]. Anticoagulation and antiplatelet therapies increase the risk of pocket hematoma, and prior research has established a connection between pocket hematoma and increased risk of device infection [13, 25].

**Fig. 3.**
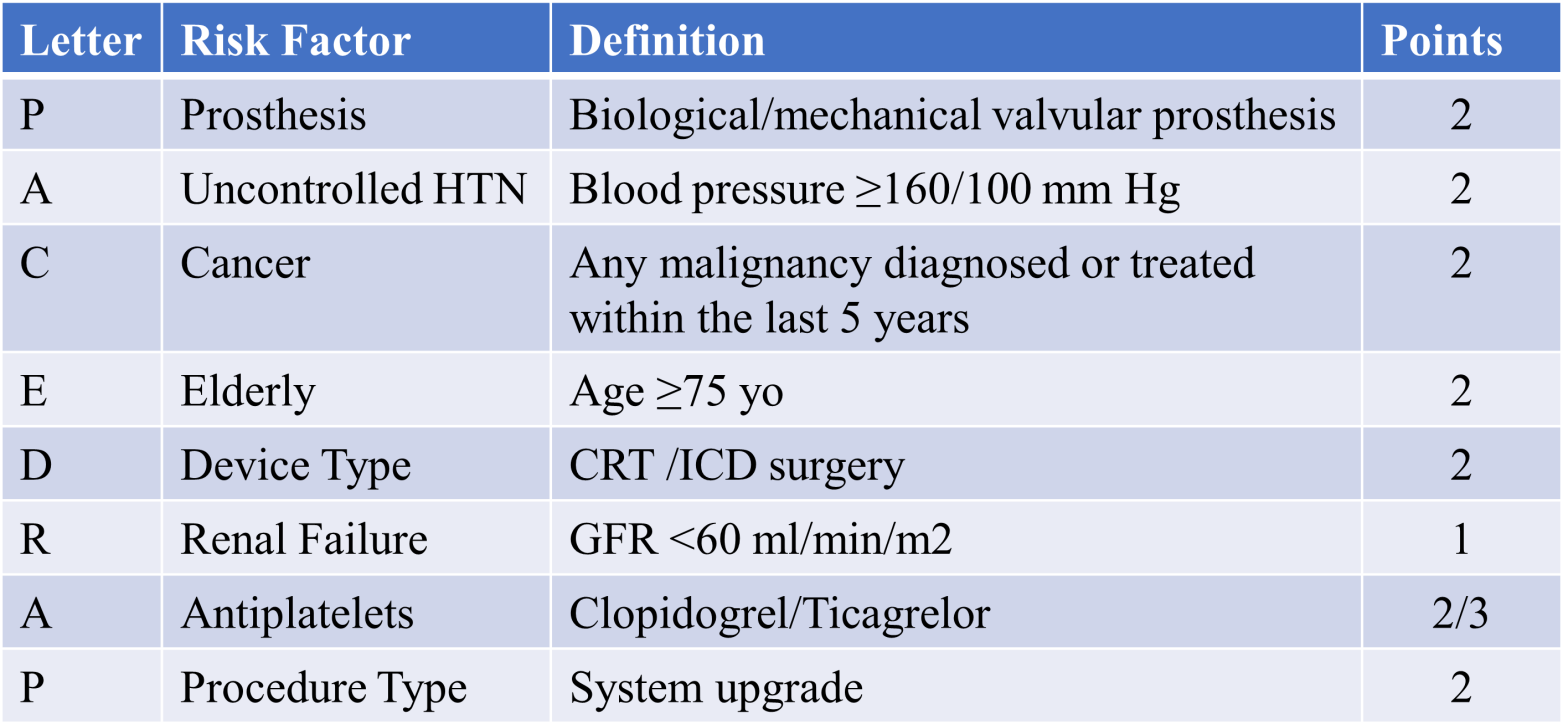
**The PACE DRAP score, used to predict the likelihood of significant bleeding complications following CIED procedures**. Abbreviations: CIED, cardiac implantable electronic device; HTN, arterial hypertension; yo, years old; CRT, cardiac resynchronization therapy; ICD, implantable cardioverter defibrillator; GFR, glomerular filtration rate.

The PACE DRAP score is an acronym that includes eight risk factors, presence of valvular prosthesis (P); uncontrolled arterial hypertension (A); cancer (C); elderly (E); device type (D); renal failure (R); antiplatelets (A); and procedure type (P); with a maximum score of 16 points (Fig. 3). It was derived from a prospective study involving 1100 patients, it was initially assessed for its predictive value of significant bleeding following CIED implantation [26]. Subsequently, the same group utilized the PACE DRAP score to evaluate the risk of CIED-related infection and discovered that it outperformed the PADIT score in discriminating between patients at high and low risk of infection [27]. A PACE DRAP score ≥6 demonstrated a sensitivity of 72.2% and specificity of 71.1% in predicting CIED infection at 1-year follow-up. Additionally, the study identified a correlation between a greater volume of pocket bleeding and a higher rate of infection [26]. However, it is worth noting that the study had limitations, including its single-center observational design, small sample size, and inclusion of only patients with ICD or CRT implants.

### 5.3 RI-AIAC (Ricerca Sulle Infezioni Associate a ImpiAnto o Sostituzione di CIED) Infection Score

In 2022, a prospective study conducted using data from the Italian RI-AIAC registry revealed significant associations between both the PADIT score and the RI-AIAC score with CIED infections, although the RI-AIAC score showed a stronger association [28]. Unlike the PADIT study, where the majority of patients received ICDs, and the PACE DRAP study, which did not include PPM patients; PPM implantation in the RI-AIAC registry represented the vast majority of the cohort and thus, it is more reflective of real-world practice [28]. Moreover, this study introduced the RI-AIAC infection score for predicting infections at 1-year follow-up and the RI-AIAC event score for predicting the clinical endpoint of infection or all-cause mortality at 1-year. The study also performed an external validation of both the infection score and event score within a cohort of 1017 patients. In this validation cohort, the RI-AIAC infection score, the same as the PADIT score, Kolek score, and Shariff score, did not demonstrate the ability to predict infections. In contrast, the RI-AIAC event score had moderate to good predictive capability for composite clinical events [28]. Different from other risk prediction scores, the RI-AIAC infection score only consists of four risks including revision/upgrading/reimplantation, CIED replacement, DM, and hospital-acquired infection, providing a more pragmatic approach for risk stratification.

### 5.4 Shariff Score

The Shariff score was developed from a retrospective single-center study as an assessment tool for peri-operative risk for patients undergoing CIED implantation [29]. The score also incorporated patient-related, device-related, as well as procedure-related factors based on previous studies. A score of more than 3 had a high predictive value for peri-procedural risk of infection at 6-month [29]. Nevertheless, when tested alongside the RI-AIAC score, PADIT score, and Kolek score in the RI-AIAC registry population, the Shariff score failed to demonstrate predictive capability for infections [28]. In the PRACTICE study, the Shariff score was used to stratify patients into low-risk and high-risk groups using a cutoff value of 3, with antibiotic prophylaxis strategies determined based on risk levels [30]. Notably, this study found no difference in the incidence of CIED-related infections between the low-risk and high-risk groups. In Diemberger’s investigation, the Shariff score also exhibited predictive value for post-transvenous lead extraction mortality, together with the presence of vegetations detected on transesophageal echocardiogram [31]. Another study focused exclusively on de novo CIED implantation and analyzed a modified Shariff score. A modified Shariff score ≥4 was proposed as an indicator of a high risk of infection following initial CIED implantation. The follow-up period in this study was 48 months, which was much longer compared to most of the previous studies proposing risk scores [32].

It is worth noting that all the discussed risk scores included device replacement or upgrade, with certain risk prediction models assigning higher weight points to this factor. For instance, the PADIT score assigned 5 points, and the RI-AIAC score assigned 2 points for revision/upgrade or reimplantation. In summary, the selection of an appropriate risk score should be guided by the specific clinical context, as there is no definitive superiority between these scores. It is advisable to choose the score that aligns most closely with the individual patient’s situation. Outstandingly, the RI-AIAC score demonstrates enhanced predictive capacity for CIED infection risk in patients with PPM, the PADIT score proves more efficacious for patients with ICDs, and the PACE DRAP score is well-suited for patients undergoing CRT or ICD implantations.

## 6. Methods to Prevent CIED Infections

### 6.1 Skin Preparation and Surgical Technique

Several preprocedural measures should be considered for preventing CIED-related infections. One of the most relevant ones is pre-operative antiseptic bathing. The Centers for Disease Control and Prevention (CDC) and the Association of Perioperative Registered Nurses (AORN) both recommend pre-operative antiseptic showering or bathing as part of infection prevention protocols for surgical procedures [33]. However, the advantages of pre-operative bathing with antiseptics in comparison to plain soap remain unclear. A meta-analysis, led by Webster, including seven trials and a total of 10,157 participants, failed to provide evidence supporting the superiority of preoperative showering or bathing with chlorhexidine over alternative wash products for reducing surgical site infections [34]. It is important to note that there has been no specific study investigating the impact of pre-operative bathing on patients planned for CIED procedures.

In contrast, antiseptic skin cleaning immediately before the procedure is proven to be crucial for reducing the presence of microorganisms on the patient’s skin. Commonly used skin antiseptics before surgery include alcoholic chlorhexidine and povidone-iodine. Existing evidence from surgical literature shows greater effectiveness of alcoholic chlorhexidine [35, 36]. Consequently, the 2020 European Heart Rhythm Association (EHRA) international consensus document on preventing, diagnosing, and treating CIED infections recommends alcoholic chlorhexidine over povidone-iodine [8]. Furthermore, effective lead management is a key pre-procedural strategy to prevent the development of CIED-related infections. The EHRA 2020 guidelines identify the presence of abandoned leads and having ≥2 leads as factors that increase the risk of infection [8]. Similarly, using non-powdered gloves is associated with a lower risk of infection by reducing local inflammation [8].

There have been no RCTs specifically designed to compare different skin antiseptics in patients undergoing CIED procedures. The consensus is that good surgical techniques can reduce the chance of surgical site infection. For CIED-related procedures, the surgical techniques can vary from the size of the incision, the choice of vascular access approach, and the selection between submuscular and subcutaneous device placement, to the method of pocket closure. A meta-analysis conducted by Atti *et al*. [37] analyzed 23 studies comparing the safety profiles of cephalic vein cut-down versus axillary or subclavian vein puncture. This analysis concluded that there was no significant difference in device infection rates between the two vascular approaches. While opting for submuscular device placement has advantages including reduced risks of device migration, skin erosion, and improved cosmetic outcomes; it requires more blunt dissection of the pectoralis muscle, which could potentially result in increased pocket trauma and hematoma. Pocket hematoma is a well-known risk factor for CIED-related infections. However, an international multicenter study involving approximately 1000 patients compared subcutaneous and submuscular approaches to ICD implantation and found no statistically significant difference in infection rates [38].

### 6.2 Antimicrobial Prophylaxis

In addition to the performance of standard-of-care aseptic techniques, the value of pre-operative antibiotic prophylaxis before CIED procedures is well acknowledged. The 2017 Heart Rhythm Society (HRS) consensus statement on the management on CIED, the 2010 American Heart Association (AHA) statement on CIED infections, the 2013 Infectious Disease Society of America (IDSA) Practice Guidelines for Antimicrobial Prophylaxis in Surgery and the 2020 EHRA international consensus document on how to prevent, diagnose, and treat CIED infections, all recommend a single dose of a cephalosporin to be administered within one hour of the surgical incision, with clindamycin and vancomycin within two hours as alternatives for patients with a ß-lactam allergy [8, 39, 40, 41]. The PADIT trial was designed to evaluate the clinical effectiveness of an incremental perioperative antibiotic approach in reducing device-related infections. This study, involving a large cohort of 19,603 patients across 28 centers, employed a cluster-randomized crossover trial design, with four randomly assigned 6-month periods during which centers implemented either conventional or incremental periprocedural antibiotic regimens for all CIED procedures [42]. Conventional antibiotic protocols were the guideline-recommended single-dose pre-procedural cefazolin or vancomycin, and the incremental strategy involved a bacitracin pocket washout in addition to post-operative cephalexin or cefadroxil. The trial observed a low infection rate (1.11%), with no statistically significant difference in infection rates between the conventional and the incremental antibiotic groups. Prominently, 12,842 patients (65.5%) were high-risk patients and most high-risk patients underwent generator change (61.6%) [42].

The PRACTICE trial adopted a risk-stratified antibiotic regimen based on the Shariff score to investigate infection prevention [30]. Patients in the low-risk group, defined by a Shariff score of <3, received two intravenous antibiotic administrations, the first administered one hour before skin incision and the second eight hours thereafter. In contrast, the high-risk group, with a Shariff score ≥3, underwent a prolonged 9-day protocol involving intravenous antibiotics one hour before skin incision, followed by additional intravenous administrations every eight hours for two days, followed by seven days of oral prophylaxis. The choice of antibiotic was guided by the microbiological analysis derived from biopsy specimens and blood cultures of CIED infections reported within the study institute. This study found no statistical difference in the CIED-related infection rates between the low-risk and high-risk groups [30]. Nevertheless, this study is subject to limitations due to its single-center, non-randomized design, and the absence of a control group implementing the two-antibiotic administration strategy for high-risk patients.

While there is clear evidence supporting the use of preoperative antibiotics, the evidence for using postoperative antibiotics is insufficient. Current guidelines do not recommend routine administration of intravenous or oral antibiotics after CIED implantation, and the HRS and AHA guidelines advise against post-operative prophylactic antibiotic use [39, 40]. Chesdachai *et al*. [43] conducted a comprehensive systematic review and meta-analysis, summarizing recent studies that aimed to re-evaluate the role of antibiotics for more than 24 hours post-procedure in preventing CIED infections. This analysis included eight studies, comprising two RCTs and six cohort studies involving a total of 26,187 patients. The findings once again demonstrated no clear benefit of postoperative antibiotics in preventing infections or reducing mortality in patients undergoing CIED implantation, replacement, or upgrade [43]. Despite these guideline recommendations and the limited evidence supporting its efficacy, post-operative antibiotic use remains a common practice in many healthcare institutions. In addition to intravenous and oral antibiotics, research into the use of topical antibiotics after wound closure has yielded inconclusive results. The current evidence suggests that there is no clear advantage of using topical antibiotics following surgical wound closure [44, 45, 46]. The use of an antibiotic pouch or envelope is discussed separately.

### 6.3 Wound Closure

The primary closure of CIED pockets employs sutures as the established standard of care. The choice of an appropriate suture for each anatomical layer plays an important role in both wound healing and cosmetic results. Typically, the smallest feasible suture that can deliver sufficient support should be used. Vicryl, characterized as a synthetic braided co-polymer suture with minimal tissue reactivity, is the most widely employed absorbable suture material for pocket closure. Additional suture materials in use include Dexon, Maxon, and Monocryl. An innovative option involves the utilization of unidirectional barbed sutures like V-Loc, which facilitates knotless stitching through the securement of sutures with built-in barbs. The efficacy and safety of this approach have been evaluated in several surgical studies [47, 48].

Typically, a three-layer wound closure method is adopted following CIED implantation. The initial layer addresses the fascia and muscle, followed by the second layer involving subcutaneous tissue, with the final layer closing the skin. The two-layer technique entails suturing the deep fascia and muscle in the first layer, providing isolation for the pocket, while the second layer is more superficial, providing a firm foundation for the overlying skin. Yao *et al*. [49] investigated the low-intensity single-layer method for CIED wound closure. In comparison with the traditional two-layer approach, revealed that the single-layer method did not result in an elevated rate of device-related infections and demonstrated a comparable rate of pocket hematoma [49].

The CIED pocket closure can be achieved with either interrupted or continuous sutures. Interrupted sutures were superior in terms of pocket hematoma formation and pocket infection. However, previous studies have demonstrated that suture techniques are not related to CIED infections [50, 51].

Besides sutures, alternative methods like staples, skin closure devices, and adhesives have been evaluated in patients who are undergoing CIED implantation [52, 53, 54, 55]. While these studies consistently report similar outcomes, their limitations lie in their single-center and observational nature, as well as their small sample sizes. Furthermore, the study populations were often limited to new implants, and the safety profile of these closure methods in patients with CIED replacement or upgrade remains unclear.

### 6.4 Pocket Irrigation

Pocket irrigation is considered an effective strategy for preventing infections in CIED procedures. Importantly, vigorous pocket irrigation has been acknowledged as crucial for eliminating damaged tissue and reducing the concentration of contaminants on the skin [8]. A range of antimicrobial irrigation solutions, spanning from antibiotic solutions to antiseptics such as povidone-iodine, chlorhexidine gluconate (CHG), hydrogen peroxide, sodium hypochlorite, acetic acid, hypochlorous acid and combined solutions can be used. Nonetheless, current evidence presents mixed results, with some studies revealing a reduction in infection rates among patients who underwent pocket irrigation, while others reported conflicting outcomes [56]. The 2010 AHA statement on CIED infections lacks clear recommendations about routine pocket irrigation during CIED procedures, while the 2020 EHRA international consensus document on how to prevent, diagnose, and treat CIED infections, recommends pocket irrigation after device and lead removal with sterile normal saline [8, 40]. Furthermore, the National Institute for Health and Care Excellence (NICE) guidelines on surgical site infection: prevention and treatment, advise against wound irrigation to reduce the risk of surgical site infection [57]. Despite the controversial clinical data and guideline recommendations, the use of antimicrobial irrigation is widely adopted in current practice as reported in an international survey [58].

Povidone-iodine is a commonly used non-antibiotic irrigation agent due to its broad antimicrobial spectrum, effectiveness against biofilms, and benign allergenic profile. It was extensively studied in patients going through breast surgery, demonstrating superiority in reducing capsular contracture and surgical site infection [59]. Likewise, the use of antiseptic irrigation during total joint arthroplasty with povidone-iodine and CHG appears to be associated with a potential reduction in the risk of periprosthetic joint infections in patients undergoing both primary and revision total hip and knee arthroplasties [60]. Nevertheless, the study comparing the use of povidone-iodine solution to saline for CIED pocket irrigation failed to demonstrate any benefit in infection prevention [61]. More recently, data from a prospective multicenter registry indicated that CHG pocket lavage significantly decreased CIED-related infections at a 1-year follow-up in patients undergoing high-risk procedures [62].

### 6.5 Capsulectomy

Capsulectomy has been suggested to decrease the risk of re-infection in patients with CIED infection undergoing device and lead extractions, with some operators advocating for capsulectomy during routine CIED replacement procedures. In theory, the decortication and removal of avascular and fibrous tissue may allow for better healing and antibiotic penetration in the pocket. Additionally, a study by Kleemann *et al*. [63] demonstrated that a third of patients experiencing revision or replacement procedures had asymptomatic bacterial colonization of the pocket, 7.5% of which went on to have a device infection with the same species [63]. However, capsulectomy carries certain risks especially hematoma formation, which itself is a risk factor for infection. A prospective randomized study conducted by Lakkireddy *et al*. [64] included 258 patients undergoing revision, extraction, or upgrade procedures and assigned patients to capsulectomy versus standard care only. In their findings, they reported no difference in the risk of superficial infections (1.5% *vs*. 4.7%, respectively; *p* = 0.13), no deep infections in either group, and a statistically significant increase in pocket hematomas with capsulectomy compared to those without it (6.1% *vs*. 0.8%; *p* = 0.03) [64]. Consequently, given the lack of evidence for its benefit, capsulectomy is not recommended during routine pocket revisions by the current EHRA international consensus guidelines [8].

### 6.6 Antibacterial Envelope

**Fig. 4.**
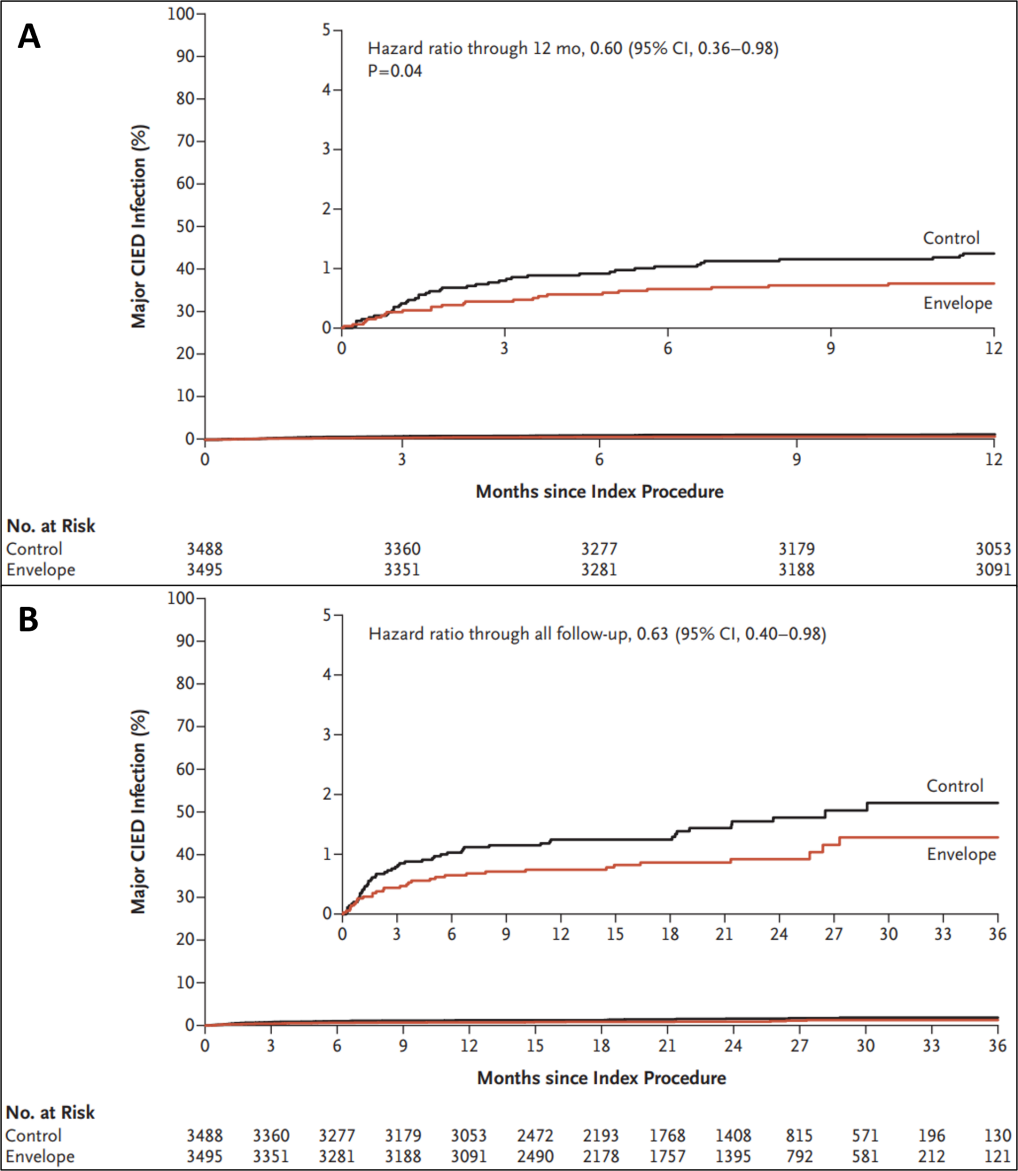
**Kaplan–Meier Curves for first major CIED infection**. Results are for the overall randomized cohort through 12 months (A) and all follow-up (B), they were not adjusted for multiple comparisons. HR is derived from Cox regressions, with stratification according to device class, and indicates the relative (envelope *vs*. control) risk of CIED infection. Reprinted from the NEJM article, by Tarakji *et al*. [65]. Antibacterial Envelope to Prevent Cardiac Implantable Device Infection, The WRAP-IT trial, with permission from the NEJM. Abbreviations: CIED, cardiac implantable electronic devices; CI, confidence interval; No. at Risk, number at risk; HR, hazard ratio.

Antibacterial envelopes (AE) have been increasingly used as a potential intervention to reduce the rates of CIED infections through the local release of antibiotics to prevent biofilm formation in the device pocket. The initial generations were not absorbable, but more recent AE generations, that release minocycline and rifampin, have become fully absorbable within nine weeks. These newer generations have been the focus of numerous trials and studies (TYRX™, Medtronic, Minneapolis, MN, US). The WRAP-IT trial randomized patients undergoing CIED replacements or upgrades or initial CRT implantations to AE versus standard-of-care infection prevention strategies [65]. In this analysis of 6983 patients, the use of AE was associated with a significantly lower incidence of major CIED infections (12-month Kaplan–Meier estimated event rate, 0.7% *vs*. 1.2%; HR 0.60; 95% CI = 0.36 to 0.98; *p* = 0.04) (Fig. 4, Ref. [65]). Similarly, a large dataset analysis of the national readmissions database revealed lower rates of CIED infections in patients who received AE compared to those who did not (1.2% *vs*. 2.2%, respectively; *p* < 0.001) [66], and several meta-analyses yielded comparable results [67, 68].

**Fig. 5.**
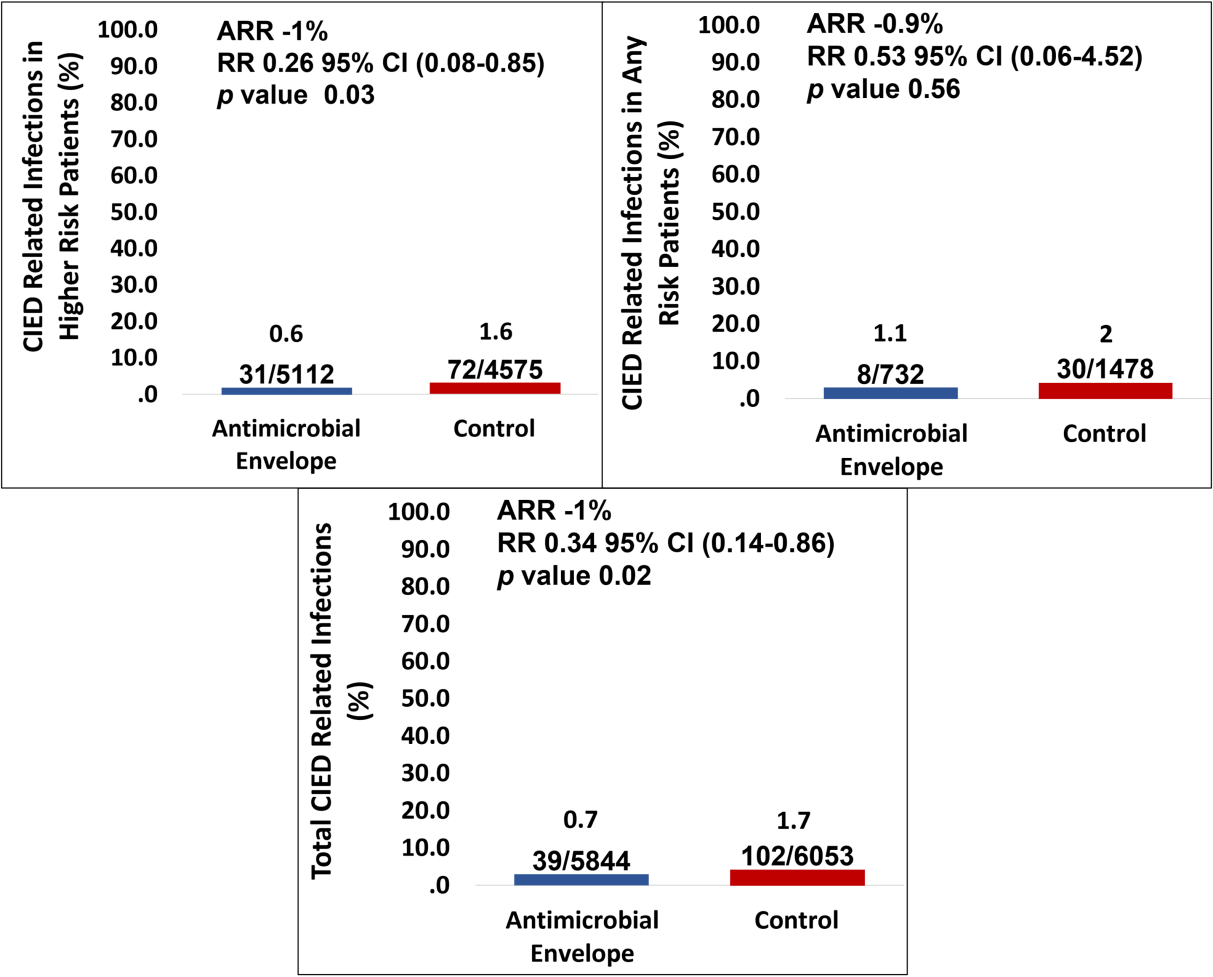
**Bar graphs showing the effect of an antibiotic envelope on major CIED-related infections according to patient risk, showing statistically significant differences in the total of patients, and in higher risk patients, without differences in any risk patients in the subgroup analysis**. Adapted from the forest plot of the Medicine article by Asbeutah *et al*. [67]. The role of an antibiotic envelope in the prevention of major cardiac implantable electronic device infections: A systematic review and meta-analysis, with permission from the Medicine journal. Abbreviations: ARR, absolute risk reduction; CIED, cardiac implantable electronic devices; CI, confidence interval; RR, risk ratio.

Particularly, patients at higher risk of infection may benefit more from the utilization of AE. In a retrospective study by Chaudhry *et al*. [69], comparing outcomes of AE to standard infection control, AE use was associated with a significantly lower risk of local infections (0 *vs*. 2.6%, respectively; *p* = 0.04), with a more pronounced difference in patients with a PADIT score >7 (0 *vs*. 9.9%, respectively; *p* = 0.01) [69]. This benefit was reproduced in subsequent studies, such as the REINFORCE project which analyzed the outcomes of 1819 patients undergoing CIED procedures (872 with AE, 947 without), and demonstrated significantly lower infection event rates in the AE group (0.8% *vs*. 2.4%, respectively; *p* = 0.007) [70]. Furthermore, a meta-analysis conducted by Asbeutah *et al*. [67] in 2020, concluded that employing an antibiotic envelope during CIED implantation significantly reduced major device-related infections, especially in patients with a higher risk of device-related infections (Fig. 5, Ref. [67]). Therefore, the 2020 EHRA international consensus document on how to prevent, diagnose, and treat CIED infections, recommends using AE in high-risk situations, such as those defined in the WRAP-IT study or those with specific patient, procedure, and device-related risk factors [8].

### 6.7 Chlorhexidine Gluconate Pocket Lavage

CHG has demonstrated rapid and potent antimicrobial and antifungal activity, effectively targeting various bacteria, and exhibiting the ability to inactivate DNA and RNA viruses [71]. Its remarkable efficacy, achieving nearly 100% effectiveness in just 30 seconds, persists for an extended period of up to 48 hours, and remains unaffected even upon contact with blood or other bodily fluids [72], making it the agent of choice for CIED-related infection prevention.

**Fig. 6.**
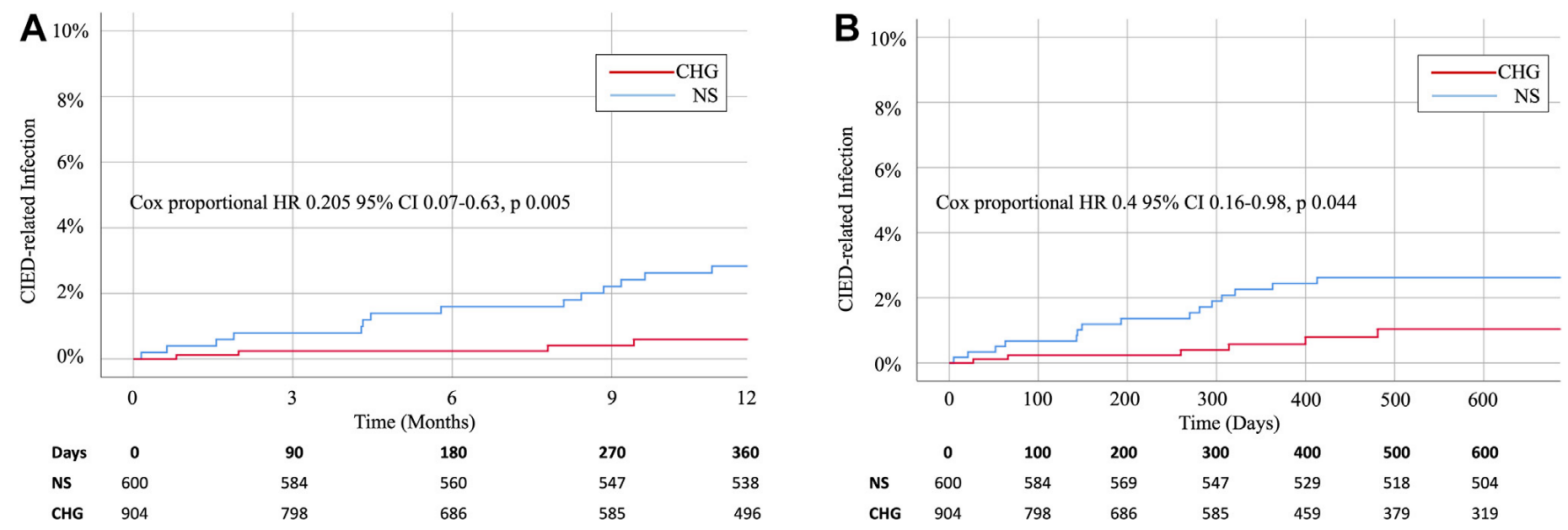
**Kaplan-Meier Curves demonstrating Cox Hazard Ratios for CHG lavage compared to NS**. (A) Primary efficacy outcome of cardiac implantable electronic device (CIED)-related infection at 1 year. (B) Secondary analysis of CIED-related infection during long-term follow-up. Reprinted from the Heart Rhythm article, by Diaz *et al*. [62]. Chlorhexidine gluconate pocket lavage to prevent cardiac implantable electronic device infection in high-risk procedures, with permission from the Heart Rhythm journal. Abbreviations: CIED, cardiac implantable electronic device; NS, normal saline; CHG, chlorhexidine gluconate; HR, hazard ratio; CI, confidence interval.

Utilizing CHG pocket lavage in high-risk procedures (such as generator changes, device upgrades, and lead or pocket revisions) has emerged as a secure and cost-effective strategy to prevent CIED infections without associated adverse events. Diaz *et al*. [62] explored the impact of CHG pocket lavage for high-risk procedures. CHG pocket lavage, involving irrigation with 20 ml of 2% CHG and normal saline, was compared to normal saline alone. At 12 months, CHG lavage resulted in significantly fewer CIED-related infections compared to the normal saline group (0.4% *vs*. 2.3%) (Fig. 6, Ref. [62]). Propensity score matching of the sample confirmed the reduction in infections with CHG (0.2% *vs*. 2.5%). In addition, there were no adverse events reported with CHG use [62]. This study opens the possibility of implementing CHG pocket lavage in high-risk procedures as a safe strategy to reduce infections. Nonetheless, it is worth noting that the CHG concentration used in this study is not widely available in a sterile format. The extrapolation of these results to the use of CHG in lower concentrations, as available in most developed countries, is yet to be determined.

## 7. Alternative Approach in High-Risk Patients

Subcutaneous ICD (S-ICD) implantation has been proposed as an alternative approach to transvenous-ICD (TV-ICD) aiming to mitigate lead-related complications and systemic infections [73]. This device is being increasingly accepted, and its use after TV-ICD extraction has grown progressively, with one study reporting an increase from 9% in 2011 to 85% in 2017. This trend may be witnessed since it does not require the insertion of any lead into the cardiovascular system, and it is particularly suitable for patients with limited venous access or at high risk of infection [74].

The advantages of S-ICD include eliminating the need for vascular access, the possibility of fluoroless implantation, the reduced mid-term risk of lead malfunction, the elimination of various procedural risks like pneumothorax and cardiac tamponade, improved arrhythmia discrimination, the relative ease of extraction, and the absence of risk for endocarditis in case of a hardware infection [75]. The PRAETORIAN trial by Knops *et al*. [76], was the first prospective RCT to compare S-ICD versus TV-ICD therapy. This study included 849 patients (426 in the S-ICD group *vs.* 423 in the TV-ICD group) and demonstrated that for patients with an indication for an ICD without pacing requirement, the S-ICD was non-inferior to the TV-ICD regarding device-related complications and inappropriate shocks (HR 0.99; 95% CI = 0.71–1.39; *p* = 0.01 for noninferiority, *p* = 0.95 for superiority). It also resulted in fewer lead-related complications with no difference in mortality [76]. Consistently, a meta-analysis by Rordorf *et al*. [73], with 13 studies comprising 9073 patients, showed no statistically significant difference in the composite outcome of device-related complications and inappropriate shocks between patients undergoing S-ICD *vs*. TV-ICD (OR 0.80; 95% CI = 0.53–1.19) [73]. Likewise, Fong *et al*. [77], demonstrated the superiority of S-ICD over TV-ICD relating to lead-related complications (risk ratio [RR] 0.14; 95% CI = 0.07–0.29; *p* < 0.0001), with comparable efficacy and safety outcomes (device-related complications RR 0.59; 95% CI = 0.33–1.04; *p* = 0.070).

Remarkably, the S-ICD can be considered a safe choice for patients who have previously had their TV-ICD removed. It is particularly favored for younger patients and in cases where extraction is necessary due to lead infection. A previous study by Viani *et al*. [74], indicated that both S-ICD and TV-ICD strategies have demonstrated comparable complication rates, with the complication rate being lower when the S-ICD generator was positioned in a sub-or intermuscular pocket.

## 8. Conclusions

Numerous strategies have been described for the prevention of CIED-related infections, having yielded positive outcomes. The medical industry has recognized this concern and has made substantial efforts to reform the landscape of CIED implantation, striving to enhance the procedure’s safety and accessibility, even for vulnerable populations, such as immunocompromised individuals, particularly within the Oncology field. Multiple risk assessment scores have been introduced to mitigate the risk of CIED infections. Nevertheless, the determination of the appropriate risk score and preventive measures remains a task that requires individualized evaluation, grounded in evidence-based medicine. It is also crucial to acknowledge that host and procedural factors, and infection pathogenesis elements must be considered in the prevention, diagnosis, and management of CIED-related infections, which constitute one of the most concerning complications linked to CIED implantation, given their potential to augment morbidity, mortality, and healthcare expenditures.

## Abbreviations

AE, Antibacterial envelopes; AORN, Association of Perioperative Registered Nurses; CRT, Cardiac Resynchronization Therapy; CRT-D, Cardiac Resynchronization Therapy - Defibrillators; CHF, Congestive heart failure; CIED, Cardiac implantable electronic devices; CDC, Centers for Disease Control and Prevention; COPD, Chronic obstructive pulmonary disease; DM, Diabetes mellitus; ESRD, End-stage renal disease; ICD, Implantable Cardioverter-Defibrillator; NIS, National Inpatient Sample; PPM, Permanent pacemakers; RCT, Randomized controlled trial.

## References

[1] Han HC, Hawkins NM, Pearman CM, Birnie DH, Krahn AD (2021). Epidemiology of cardiac implantable electronic device infections: incidence and risk factors. *Europace*.

[2] Borleffs CJW, Thijssen J, de Bie MK, van Rees JB, van Welsenes GH, van Erven L (2010). Recurrent implantable cardioverter-defibrillator replacement is associated with an increasing risk of pocket-related complications. *Pacing and Clinical Electrophysiology*.

[3] Poole JE, Gleva MJ, Mela T, Chung MK, Uslan DZ, Borge R (2010). Complication rates associated with pacemaker or implantable cardioverter-defibrillator generator replacements and upgrade procedures: results from the REPLACE registry. *Circulation*.

[4] Greenspon AJ, Patel JD, Lau E, Ochoa JA, Frisch DR, Ho RT (2011). 16-year trends in the infection burden for pacemakers and implantable cardioverter-defibrillators in the United States 1993 to 2008. *Journal of the American College of Cardiology*.

[5] Olsen T, Jørgensen OD, Nielsen JC, Thøgersen AM, Philbert BT, Johansen JB (2019). Incidence of device-related infection in 97 750 patients: clinical data from the complete Danish device-cohort (1982-2018). *European Heart Journal*.

[6] Joy PS, Kumar G, Poole JE, London B, Olshansky B (2017). Cardiac implantable electronic device infections: Who is at greatest risk. *Heart Rhythm*.

[7] Modi V, Shah K, Ferraro B, Gasimli-Gamache L, Nanda S, Stevens S (2023). Cardiac implantable electronic device implantation and device-related infection. *Europace*.

[8] Blomström-Lundqvist C, Traykov V, Erba PA, Burri H, Nielsen JC, Bongiorni MG (2020). European Heart Rhythm Association (EHRA) international consensus document on how to prevent, diagnose, and treat cardiac implantable electronic device infections-endorsed by the Heart Rhythm Society (HRS), the Asia Pacific Heart Rhythm Society (APHRS), the Latin American Heart Rhythm Society (LAHRS), International Society for Cardiovascular Infectious Diseases (ISCVID) and the European Society of Clinical Microbiology and Infectious Diseases (ESCMID) in collaboration with the European Association for Cardio-Thoracic Surgery (EACTS). *Europace*.

[9] Bongiorni MG, Tascini C, Tagliaferri E, Di Cori A, Soldati E, Leonildi A (2012). Microbiology of cardiac implantable electronic device infections. *Europace*.

[10] Uslan DZ, Sohail MR, St Sauver JL, Friedman PA, Hayes DL, Stoner SM (2007). Permanent pacemaker and implantable cardioverter defibrillator infection: a population-based study. *Archives of Internal Medicine*.

[11] Fey PD, Olson ME (2010). Current concepts in biofilm formation of Staphylococcus epidermidis. *Future Microbiology*.

[12] Howden BP, Giulieri SG, Wong Fok Lung T, Baines SL, Sharkey LK, Lee JYH (2023). Staphylococcus aureus host interactions and adaptation. *Nature Reviews. Microbiology*.

[13] Polyzos KA, Konstantelias AA, Falagas ME (2015). Risk factors for cardiac implantable electronic device infection: a systematic review and meta-analysis. *Europace*.

[14] Tompkins C, McLean R, Cheng A, Brinker JA, Marine JE, Nazarian S (2011). End-stage renal disease predicts complications in pacemaker and ICD implants. *Journal of Cardiovascular Electrophysiology*.

[15] Bloom H, Heeke B, Leon A, Mera F, Delurgio D, Beshai J (2006). Renal insufficiency and the risk of infection from pacemaker or defibrillator surgery. *Pacing and Clinical Electrophysiology*.

[16] Hercé B, Nazeyrollas P, Lesaffre F, Sandras R, Chabert JP, Martin A (2013). Risk factors for infection of implantable cardiac devices: data from a registry of 2496 patients. *Europace*.

[17] de Vries FEE, Gans SL, Solomkin JS, Allegranzi B, Egger M, Dellinger EP (2017). Meta-analysis of lower perioperative blood glucose target levels for reduction of surgical-site infection. *The British Journal of Surgery*.

[18] Khalil M, Karimzad K, Durand JB, Malek AE, Raad II, Viola GM (2020). Prevention of Cardiac Implantable Electronic Device-Related Infection in Patients With Cancer: The Role of a Comprehensive Prophylactic Bundle Approach That Includes the Antimicrobial Mesh. *Open Forum Infectious Diseases*.

[19] Prutkin JM, Reynolds MR, Bao H, Curtis JP, Al-Khatib SM, Aggarwal S (2014). Rates of and factors associated with infection in 200 909 Medicare implantable cardioverter-defibrillator implants: results from the National Cardiovascular Data Registry. *Circulation*.

[20] Birnie DH, Healey JS, Wells GA, Verma A, Tang AS, Krahn AD (2013). Pacemaker or defibrillator surgery without interruption of anticoagulation. *The New England Journal of Medicine*.

[21] Tarakji KG, Korantzopoulos P, Philippon F, Biffi M, Mittal S, Poole JE (2021). Infectious consequences of hematoma from cardiac implantable electronic device procedures and the role of the antibiotic envelope: A WRAP-IT trial analysis. *Heart Rhythm*.

[22] Darouiche RO (2001). Device-associated infections: a macroproblem that starts with microadherence. *Clinical Infectious Diseases*.

[23] Birnie DH, Wang J, Alings M, Philippon F, Parkash R, Manlucu J (2019). Risk Factors for Infections Involving Cardiac Implanted Electronic Devices. *Journal of the American College of Cardiology*.

[24] Ahmed FZ, Blomström-Lundqvist C, Bloom H, Cooper C, Ellis C, Goette A (2021). Use of healthcare claims to validate the Prevention of Arrhythmia Device Infection Trial cardiac implantable electronic device infection risk score. *Europace*.

[25] Essebag V, Verma A, Healey JS, Krahn AD, Kalfon E, Coutu B (2016). Clinically Significant Pocket Hematoma Increases Long-Term Risk of Device Infection: BRUISE CONTROL INFECTION Study. *Journal of the American College of Cardiology*.

[26] Sławek-Szmyt S, Araszkiewicz A, Grygier M, Szmyt K, Seniuk W, Waśniewski M (2020). PACE DRAP: a simple score for predicting significant bleeding complications after cardiac implantable electronic device surgery. *Polish Archives of Internal Medicine*.

[27] Sławek-Szmyt S, Araszkiewicz A, Grygier M, Szmyt K, Chmielewska-Michalak L, Seniuk W (2020). Predictors of Long-Term Infections After Cardiac Implantable Electronic Device Surgery - Utility of Novel PADIT and PACE DRAP Scores. *Circulation Journal*.

[28] Boriani G, Proietti M, Bertini M, Diemberger I, Palmisano P, Baccarini S (2022). Incidence and Predictors of Infections and All-Cause Death in Patients with Cardiac Implantable Electronic Devices: The Italian Nationwide RI-AIAC Registry. *Journal of Personalized Medicine*.

[29] Shariff N, Eby E, Adelstein E, Jain S, Shalaby A, Saba S (2015). Health and Economic Outcomes Associated with Use of an Antimicrobial Envelope as a Standard of Care for Cardiac Implantable Electronic Device Implantation. *Journal of Cardiovascular Electrophysiology*.

[30] Malagù M, Vitali F, Brieda A, Cimaglia P, De Raffele M, Tazzari E (2022). Antibiotic prophylaxis based on individual infective risk stratification in cardiac implantable electronic device: the PRACTICE study. *Europace*.

[31] Diemberger I, Migliore F, Biffi M, Cipriani A, Bertaglia E, Lorenzetti S (2018). The “Subtle” connection between development of cardiac implantable electrical device infection and survival after complete system removal: An observational prospective multicenter study. *International Journal of Cardiology*.

[32] Balla C, Brieda A, Righetto A, Vitali F, Malagù M, Cultrera R (2020). Predictors of infection after “de novo” cardiac electronic device implantation. *European Journal of Internal Medicine*.

[33] Mangram AJ, Horan TC, Pearson ML, Silver LC, Jarvis WR (1999). Guideline for Prevention of Surgical Site Infection, 1999. Centers for Disease Control and Prevention (CDC) Hospital Infection Control Practices Advisory Committee. *American Journal of Infection Control*.

[34] Webster J, Osborne S (2015). Preoperative bathing or showering with skin antiseptics to prevent surgical site infection. *Cochrane Database of Systematic Reviews*.

[35] Dumville JC, McFarlane E, Edwards P, Lipp A, Holmes A, Liu Z (2015). Preoperative skin antiseptics for preventing surgical wound infections after clean surgery. *The Cochrane Database of Systematic Reviews*.

[36] Darouiche RO, Wall MJ, Itani KMF, Otterson MF, Webb AL, Carrick MM (2010). Chlorhexidine-Alcohol versus Povidone-Iodine for Surgical-Site Antisepsis. *The New England Journal of Medicine*.

[37] Atti V, Turagam MK, Garg J, Koerber S, Angirekula A, Gopinathannair R (2020). Subclavian and Axillary Vein Access Versus Cephalic Vein Cutdown for Cardiac Implantable Electronic Device Implantation: A Meta-Analysis. *JACC. Clinical Electrophysiology*.

[38] Gold MR, Peters RW, Johnson JW, Shorofsky SR (1996). Complications associated with pectoral cardioverter-defibrillator implantation: comparison of subcutaneous and submuscular approaches. Worldwide Jewel Investigators. *Journal of the American College of Cardiology*.

[39] Indik JH, Gimbel JR, Abe H, Alkmim-Teixeira R, Birgersdotter-Green U, Clarke GD (2017). 2017 HRS expert consensus statement on magnetic resonance imaging and radiation exposure in patients with cardiovascular implantable electronic devices. *Heart Rhythm*.

[40] Baddour LM, Epstein AE, Erickson CC, Knight BP, Levison ME, Lockhart PB (2010). Update on cardiovascular implantable electronic device infections and their management: a scientific statement from the American Heart Association. *Circulation*.

[41] Bratzler DW, Dellinger EP, Olsen KM, Perl TM, Auwaerter PG, Bolon MK (2013). Clinical practice guidelines for antimicrobial prophylaxis in surgery. *American Journal of Health-system Pharmacy*.

[42] Krahn AD, Longtin Y, Philippon F, Birnie DH, Manlucu J, Angaran P (2018). Prevention of Arrhythmia Device Infection Trial: The PADIT Trial. *Journal of the American College of Cardiology*.

[43] Chesdachai S, Go JR, Hassett LC, Baddour LM, DeSimone DC (2022). The utility of postoperative systemic antibiotic prophylaxis following cardiovascular implantable electronic device implantation: A systematic review and meta-analysis. *Pacing and Clinical Electrophysiology*.

[44] Khalighi K, Aung TT, Elmi F (2014). The role of prophylaxis topical antibiotics in cardiac device implantation. *Pacing and Clinical Electrophysiology*.

[45] Heal CF, Banks JL, Lepper P, Kontopantelis E, van Driel ML (2017). Meta-analysis of randomized and quasi-randomized clinical trials of topical antibiotics after primary closure for the prevention of surgical-site infection. *The British Journal of Surgery*.

[46] Chen PJ, Hua YM, Toh HS, Lee MC (2021). Topical antibiotic prophylaxis for surgical wound infections in clean and clean-contaminated surgery: a systematic review and meta-analysis. *BJS Open*.

[47] Krishnamoorthy B, Shepherd N, Critchley WR, Nair J, Devan N, Nasir A (2016). A randomized study comparing traditional monofilament knotted sutures with barbed knotless sutures for donor leg wound closure in coronary artery bypass surgery. *Interactive Cardiovascular and Thoracic Surgery*.

[48] Rubin JP, Hunstad JP, Polynice A, Gusenoff JA, Schoeller T, Dunn R (2014). A multicenter randomized controlled trial comparing absorbable barbed sutures versus conventional absorbable sutures for dermal closure in open surgical procedures. *Aesthetic Surgery Journal*.

[49] Yao T, Nie P, Sun J, Jin Y, Zang M, Zhou S (2021). Cardiac device implant wound closure with a novel low-density suture spacing single layer method. *Pacing and Clinical Electrophysiology*.

[50] Datta G (2020). Pacemaker pocket infection rate and suture technique. *Turk Kardiyoloji Dernegi Arsivi: Turk Kardiyoloji Derneginin Yayin Organidir*.

[51] Monga IS (2021). Single Operator Observational Study of Incidence of Pocket Site Infection and Safety of Absorbable Sutures for Pocket Closure of Cardiac Implantable Electronic Devices. *Interventional Cardiology*.

[52] De Maria E (2015). New skin closure system facilitates wound healing after cardiovascular implantable electronic device surgery. *World Journal of Clinical Cases*.

[53] Lalani GG, Schricker AA, Salcedo J, Hebsur S, Hsu J, Feld G (2016). Cardiac Device Implant Skin Closure with a Novel Adjustable, Coaptive Tape-Based Device. *Pacing and Clinical Electrophysiology*.

[54] Malik J, Javed N, Rana G, Shoaib M, Ishaq U, Chauhan H (2021). Outcomes of intracutaneous sutures in comparison with intracutaneous staples in cardiac implantable-electronic device pocket closure. *Anatolian Journal of Cardiology*.

[55] Koerber SM, Loethen T, Turagam M, Payne J, Weachter R, Flaker G (2019). Noninvasive tissue adhesive for cardiac implantable electronic device pocket closure: the TAPE pilot study. *Journal of Interventional Cardiac Electrophysiology*.

[56] Rodriguez D, Thurber CJ, Romero JE, Sauer WH, Kapur S, Tadros TM (2022). Device Pocket Antibiotic Irrigation Provides No CIED-Pocket Infection Prophylaxis Advantage Compared to Normal Saline. *JACC. Clinical Electrophysiology*.

[57] National Institute for Health and Care Excellence (2006). Surgical Site Infections: Prevention and Treatment. https://www.nice.org.uk/guidance/ng125/resources/surgical-site-infections-prevention-and-treatment-pdf-66141660564421.

[58] Zheng Q, Di Biase L, Ferrick KJ, Gross JN, Guttenplan NA, Kim SG (2018). Use of antimicrobial agent pocket irrigation for cardiovascular implantable electronic device infection prophylaxis: Results from an international survey. *Pacing and Clinical Electrophysiology*.

[59] Saeg F, Schoenbrunner AR, Janis JE (2021). Evidence-Based Wound Irrigation: Separating Fact from Fiction. *Plastic and Reconstructive Surgery*.

[60] Wood T, Ekhtiari S, Mundi R, Citak M, Sancheti PK, Guerra-Farfan E (2020). The Effect of Irrigation Fluid on Periprosthetic Joint Infection in Total Hip and Knee Arthroplasty: A Systematic Review and Meta-Analysis. *Cureus*.

[61] Lakkireddy D, Valasareddi S, Ryschon K, Basarkodu K, Rovang K, Mohiuddin SM (2005). The impact of povidone-iodine pocket irrigation use on pacemaker and defibrillator infections. *Pacing and Clinical Electrophysiology*.

[62] Diaz JC, Braunstein ED, Cañas F, Duque M, Marín JE, Aristizabal J (2023). Chlorhexidine gluconate pocket lavage to prevent cardiac implantable electronic device infection in high-risk procedures. *Heart Rhythm*.

[63] Kleemann T, Becker T, Strauss M, Dyck N, Weisse U, Saggau W (2010). Prevalence of bacterial colonization of generator pockets in implantable cardioverter defibrillator patients without signs of infection undergoing generator replacement or lead revision. *Europace*.

[64] Lakkireddy D, Pillarisetti J, Atkins D, Biria M, Reddy M, Murray C (2015). IMpact of pocKet rEvision on the rate of InfecTion and other CompLications in patients rEquiring pocket mAnipulation for generator replacement and/or lead replacement or revisioN (MAKE IT CLEAN): A prospective randomized study. *Heart Rhythm*.

[65] Tarakji KG, Mittal S, Kennergren C, Corey R, Poole JE, Schloss E (2019). Antibacterial Envelope to Prevent Cardiac Implantable Device Infection. *The New England Journal of Medicine*.

[66] Shatla I, Mujer M, Baker P, Kashlan B, Pieters Z, Elkaryoni A (2022). Contemporary Trends of Antibacterial Envelope Use to Prevent Cardiac Implantable Device Infection: Insights From the National Readmission Database. *Circulation*.

[67] Asbeutah AAA, Salem MH, Asbeutah SA, Abu-Assi MA (2020). The role of an antibiotic envelope in the prevention of major cardiac implantable electronic device infections: A systematic review and meta-analysis. *Medicine*.

[68] Pranata R, Tondas AE, Vania R, Yuniadi Y (2019). Antibiotic envelope is associated with reduction in cardiac implantable electronic devices infections especially for high-power device-Systematic review and meta-analysis. *Journal of Arrhythmia*.

[69] Chaudhry U, Borgquist R, Smith JG, Mörtsell D (2022). Efficacy of the antibacterial envelope to prevent cardiac implantable electronic device infection in a high-risk population. *Europace*.

[70] Ziacchi M, Biffi M, Iacopino S, di Silvestro M, Marchese P, Miscio F (2023). REducing INFectiOns thRough Cardiac device Envelope: insight from real world data. The REINFORCE project. *Europace*.

[71] Poppolo Deus F, Ouanounou A (2022). Chlorhexidine in Dentistry: Pharmacology, Uses, and Adverse Effects. *International Dental Journal*.

[72] Schmidt K, Estes C, McLaren A, Spangehl MJ (2018). Chlorhexidine Antiseptic Irrigation Eradicates Staphylococcus epidermidis From Biofilm: An In Vitro Study. *Clinical Orthopaedics and Related Research*.

[73] Rordorf R, Casula M, Pezza L, Fortuni F, Sanzo A, Savastano S (2021). Subcutaneous versus transvenous implantable defibrillator: An updated meta-analysis. *Heart Rhythm*.

[74] Viani S, Migliore F, Tola G, Pisanò ECL, Russo AD, Luzzi G (2019). Use and outcomes of subcutaneous implantable cardioverter-defibrillator (ICD) after transvenous ICD extraction: An analysis of current clinical practice and a comparison with transvenous ICD reimplantation. *Heart Rhythm*.

[75] Lewis GF, Gold MR (2016). Safety and Efficacy of the Subcutaneous Implantable Defibrillator. *Journal of the American College of Cardiology*.

[76] Knops RE, Olde Nordkamp LRA, Delnoy PPHM, Boersma LVA, Kuschyk J, El-Chami MF (2020). Subcutaneous or Transvenous Defibrillator Therapy. *The New England Journal of Medicine*.

[77] Fong KY, Ng CJR, Wang Y, Yeo C, Tan VH (2022). Subcutaneous Versus Transvenous Implantable Defibrillator Therapy: A Systematic Review and Meta-Analysis of Randomized Trials and Propensity Score-Matched Studies. *Journal of the American Heart Association*.

